# Supplementary material for: High-speed laser writing of structural colors for full-color inkless printing
Source: Nat Commun. 2023 Feb 2;14:565. doi: 10.1038/s41467-023-36275-9 (PMC9894925; doi:10.1038/s41467-023-36275-9)
Supplement: Supplementary file 1 — supplementary information [file 41467_2023_36275_MOESM1_ESM.pdf]

Supplementary Information

High-speed laser writing of structural colors for full-color inkless printing

Jiao Geng[1,2,] Liye Xu[1,2,] Wei Yan[1,2,] Liping Shi[1,2*] and Min Qiu[1,2*]

[1]Key Laboratory of 3D Micro/Nano Fabrication and Characterization of Zhejiang Province, School of Engineering, Westlake University, 18 Shilongshan Road, Hangzhou 310024, Zhejiang Province, China.

[2]Institute of Advanced Technology, Westlake Institute for Advanced Study, 18 Shilongshan Road, Hangzhou 310024, Zhejiang Province, China.

*Corresponding author(s):

shiliping@westlake.edu.cn; qiumin@westlake.edu.cn

Supplementary Note 1: Refractive index of TiN and TiAlN

We have measured the refractive indices of our deposited TiN and TiAlN films by a variable angle spectroscopic ellipsometer (Woollam), as shown in Fig. 1. The results reveal that TiAlN manifests as a highly lossy dielectric material, whose Im (ε) is large in the visible spectral ranges. The screened plasma wavelength of TiN is measured to be at ~600 nm. Its large negative values of Re (ε) above 600 nm indicate that TiN is highly metallic in the near-infrared regime.

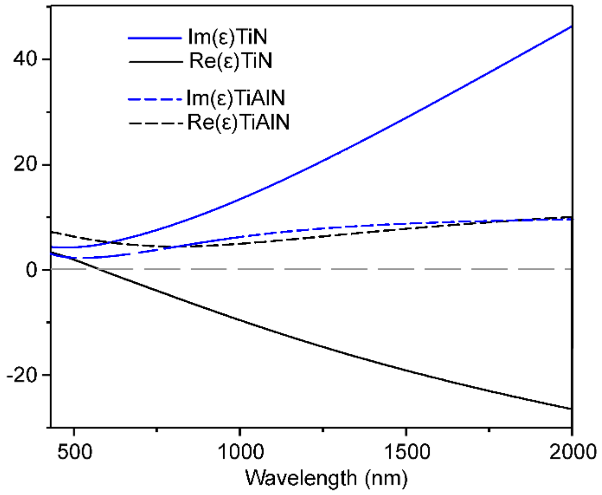

Supplementary Fig. 1 Material permittivities. Experimentally measured dielectric constants of TiN and TiAlN that are deposited by RF magnetron reactive sputtering. Source data is available.

Supplementary Note 2: Laser-induced periodic surface structures (LIPSS)

LIPSS is also called as nanoripples, a universal effect that can be observed on almost any material after the irradiation by linearly polarized lasers, particularly when using ultrashort pulses.[1] LIPSS is generally produced by far-field interference between incident light and scattered surface waves associated with near-field enhancement in vicinity of surface scatters.[2] As shown by FDTD-based numerical simulations in Fig. 2(a), when the incident light is linearly polarized, the orientation of near-field enhancement and far-field interfering patterns are perpendicular to incident light polarization (E_0). As a

result, nanoripples with orientation perpendicular to laser polarization are observed in experiments, as shown in Fig. 2(c). Instead, when circular polarization is utilized, the field enhancement is isotropic in each direction (Fig. 2b), as a result, nanoripples are not observed (Fig. 2d).

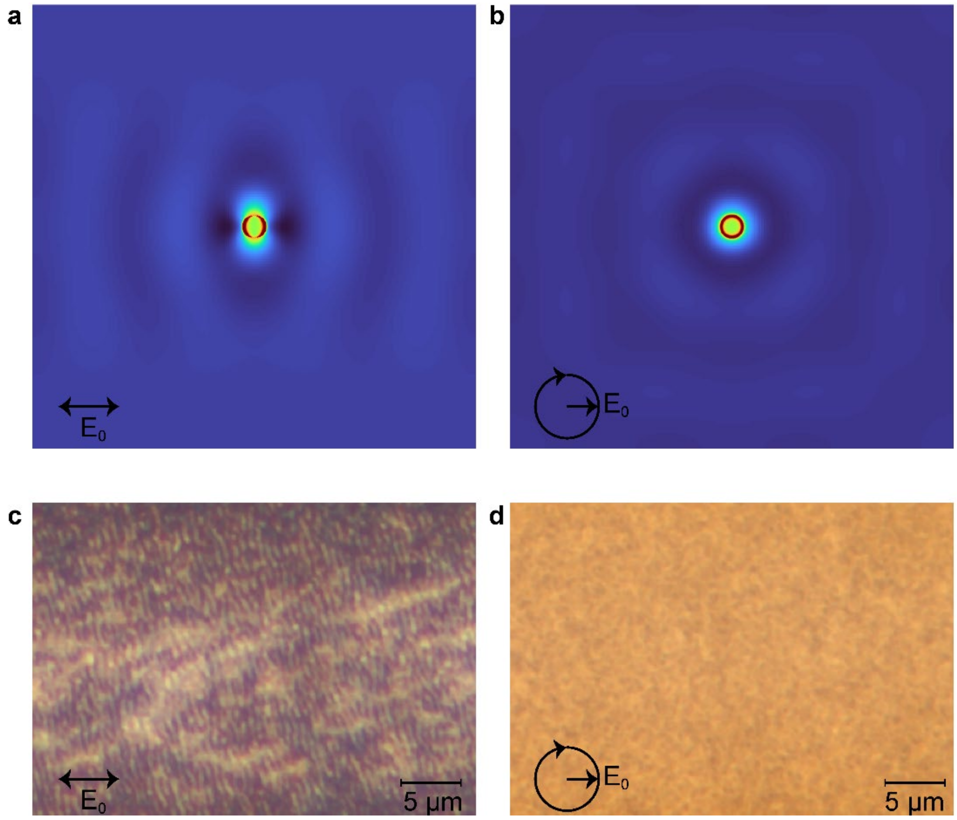

Supplementary Fig. 2 LIPSS mechanisms. (a, b) FDTD-based numerical simulation of electric field distribution at TiAlN–air interface in the presence of a cylindrical alumina nanoparticle with diameter of 300 nm and height of 100 nm. Half of the alumina particle is immersed into the TiAlN layer. The light source is a plane wave at 1030 nm with linear (a) and circular (b) polarization. Optical microscope images of laser-modified TiAlN surfaces by linear (c) and circular (d) polarization while at the same fluences and scanning speeds. The thickness of TiAlN and TiN are both 50 nm in simulations and experiments.

Supplementary Note 3: High printing spatial resolution

In order to investigate the spatial resolution of our high-speed laser printing setup, we have measured the sizes of structural colors that are written by line scanning and single spots, as shown in Fig. 3(a, b). The spot size is measured

to be 70 μm, corresponding to a printing resolution of 25400/70=360 dpi.

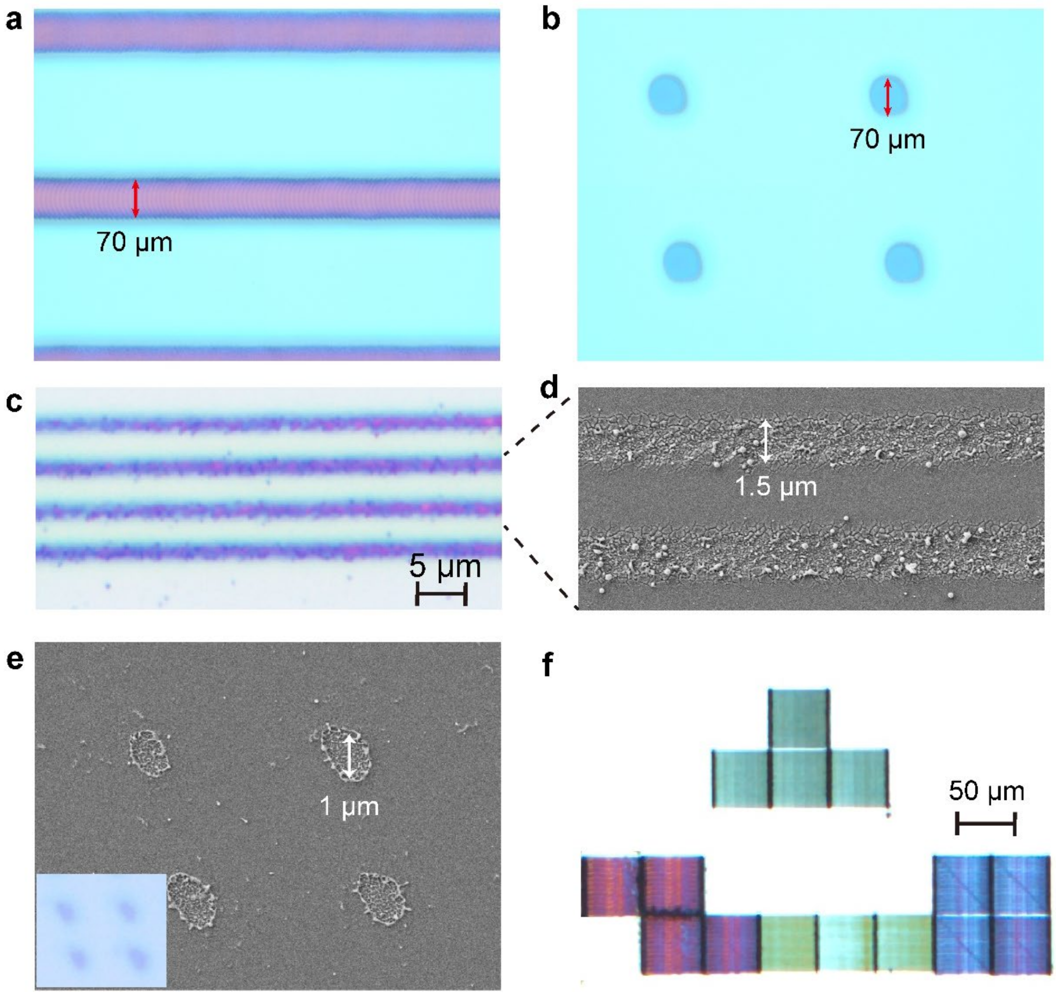

Supplementary Fig. 3 Printing resolution. Structural colors that are generated by line scanning (a) and single spots (b) when the laser beam is loosely focused by a lens with focal length of 20 mm. (c) Structural color produced by a tightly focused laser beam. Optical microscopy images and corresponding SEM images that are generated by line scanning (c, d) and single spot (e) when the laser beam is tightly focused by an objective lens with NA = 0.9. (f) optical microscopy images of color swatches (Tetris game) that are produced by meander scanning.

The resolution can be improved by utilizing a smaller laser beam spot. For instance, when focusing the laser beam by an objective lens (NA = 0.9), the printing resolution exceeds 10^4 dpi (Fig. 3c-3e). The spot sizes of pigments are generally on the order of 25 μm, resulting in a resolution of ~10^3 dpi. Therefore, our laser-printing ink-free structural colors can have one order of magnitude higher resolution than the conventional printers. The SEM images in Fig. 3(d, e) show nanoparticles depositing on cracked surface, in agreement with that

are produced by loosely focused beam.

However, we would like to clarify that, the laser beam was focused by an objective lens with very high NA for high-resolution coloring, the working distance and laser Rayleigh length are very short, but we do not have an automatic real-time focus control system for laser processing. Therefore, the system is highly sensitive to mechanical vibration, surface relief and so on. As a result, the uniformity of colors over large areas is not excellent, as shown in Fig. 3(f). Nevertheless, the resolution is not the most critical performance, because a printing resolution of 360 dpi by using loosely focusing is generally acceptable for many practical applications.

Supplementary Note 4: Reflectance at different incident angles

Fig. 4 depicts the specular reflectance spectra of a 4 cm×4 cm color swatch at different angles. The spectral profiles and the reflected peaks are nearly unchanged from 6° to 60°, suggesting that the Hue values of thin-film absorber-based structural colors are rather insensitive to the viewing angles. This is because the dielectric coatings are much thinner than the wavelength of visible light, the phase accumulation due to the propagation through the film is little. In contrast, the non-trivial phase shift at the lossy dielectric interfaces gives rise to an additional phase for interference.

Please note that a collimated light beam incident from a specific angle was used for measuring the specular reflectance spectra, therefore, the reflection intensity varies with the incident angles. However, in a real environment, the ambient room light simultaneously illuminates the samples from different angles. Therefore, the visual brightness does not obviously change with viewing angles, as confirmed by the photographs in the main text (Fig. 2f-2j).

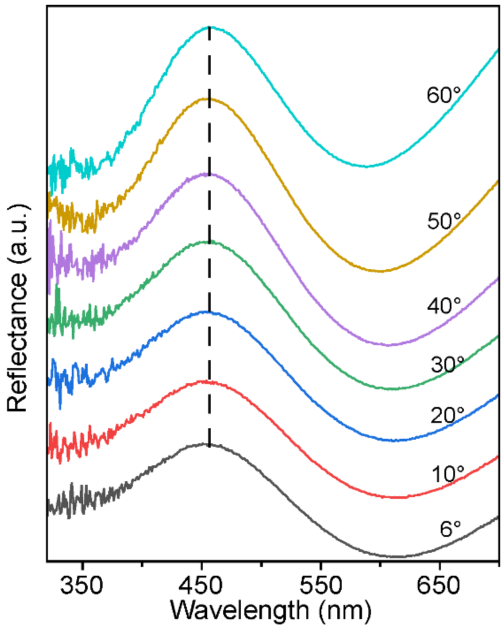

Supplementary Fig. 4 Viewing angle-independent structural colors. Reflectance spectra versus incident angles of a representative color swatch. Source data is available.

Supplementary Note 5: Large-scale EDX maps of different color swatches

Fig. 5(a-e) list the two-dimensional large-scale EDX maps of various elements and corresponding colors of the laser-written areas (left-bottom areas in the SEM images). The comparison of EDX between laser-modified areas and the pristine films confirm that all generated colors are indeed related to laser-induced oxidation, that is, nitrogen has been partially replaced by oxygen. With regard to blue, red and yellow colors that occur at high totally accumulated laser fluence, we find that the EDX signal of aluminum is reduced. This indicates that a part of aluminum has been ablated at high accumulated fluence. As a result, the EDX intensity of silicon substrate is enhanced within the laser-irradiated areas. Indeed, the ablation occurs at high accumulated fluence was also confirmed by the FIB-based cross-sectional view, as discussed in the manuscript (Fig. 4f-4j).

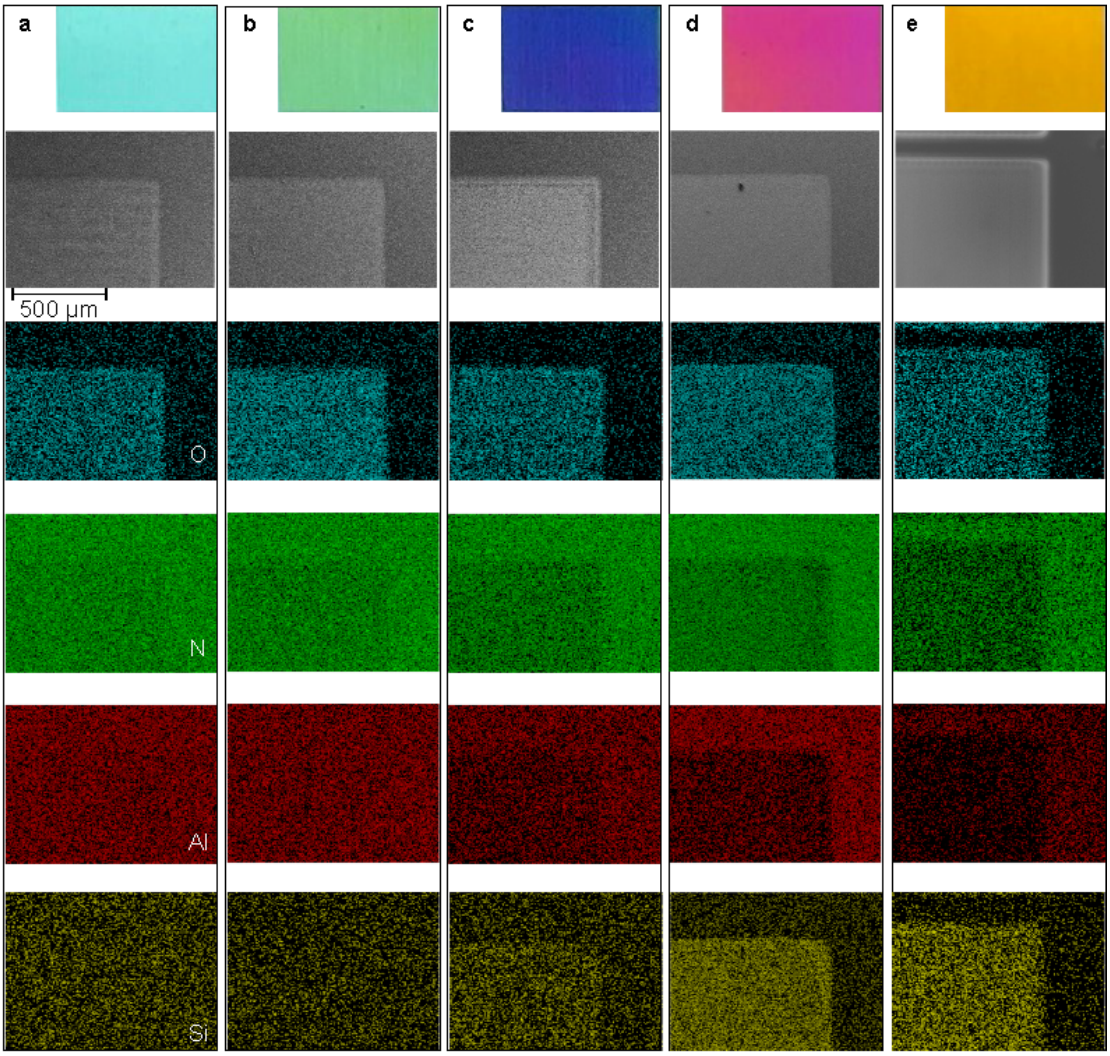

Supplementary Fig. 5 Material analysis. Photographs, Low-magnification SEM images (second row) and corresponding EDX maps (third row for oxygen; fourth row for nitrogen; fifth row for aluminum and last row for silicon) of five different color swatches (first row): **a,** light blue; **b,** green; **c,** blue; **d,** red; **e,** yellow. The laser modified areas locate in the left-bottom regions in each SEM image.

We further obtain high-magnification EDX maps of the laser-written areas, as shown in Fig. 6. We find that the dominant components of the redeposited ablative nanoparticles on surface is aluminium oxide.

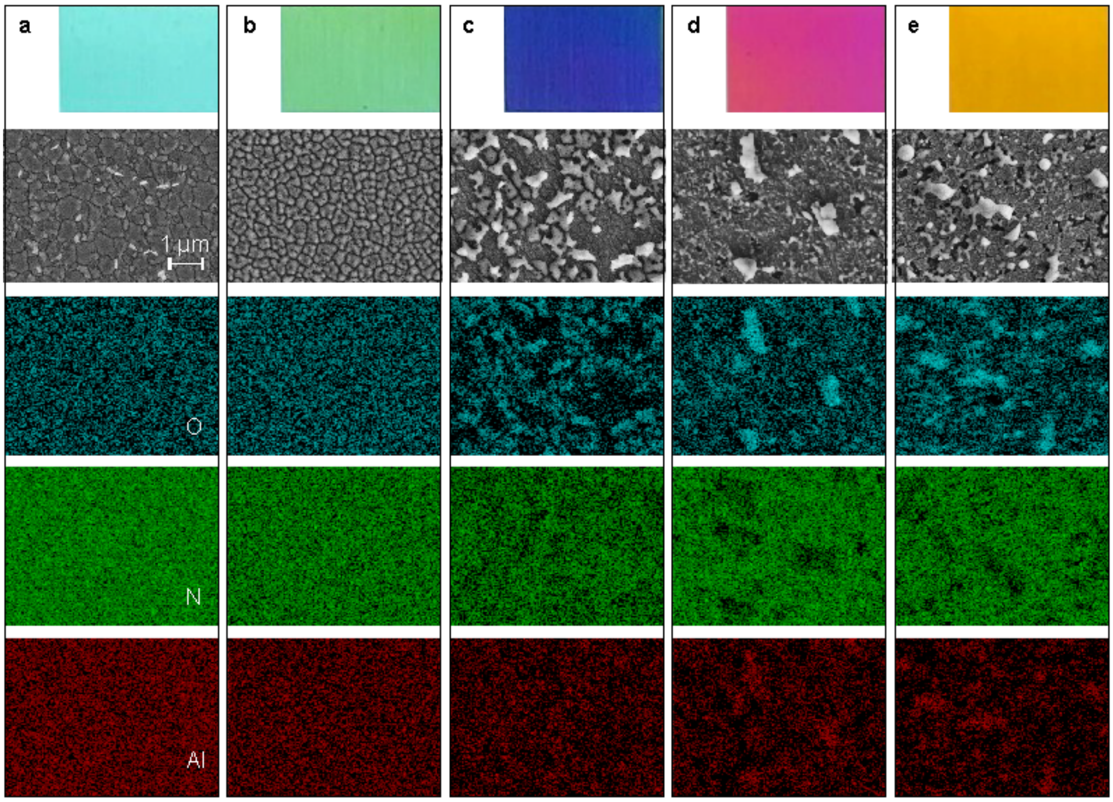

Supplementary Fig. 6 Material analysis. High-magnification SEM images (second row) and corresponding EDX maps (third row for oxygen; fourth row for nitrogen and fifth row for aluminum) of five different color palettes (first row): **a,** light blue; **b,** green; **c,** blue; **d,** red; **e,** yellow.

Supplementary Note 6: Influence of surface roughness in reflected colors

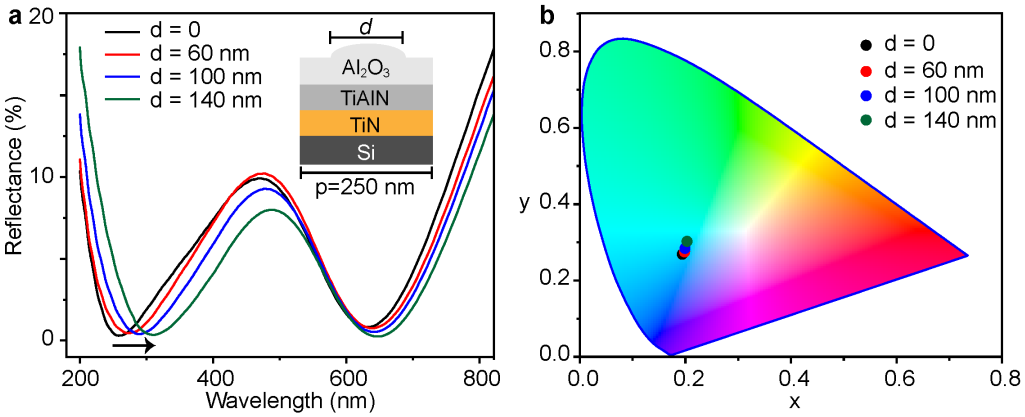

Supplementary Fig. 7 Surface roughness versus colors. (a) Simulated reflectance spectra of hybrid films when existing different diameters of alumina nanoparticles. Inset: scheme of the geometry for numerical simulation. (b)

Numerically simulated CIE 1931 color coordinates for different diameters of alumina nanoparticles on the surface of a Al2O3-TiAlN-TiN hybrid film. Source data is available.

As confirmed by the high-resolution SEM images and AFM images, the different laser-written colors exhibit different surface roughness. We investigate the influence of surface roughness in the reflected spectra by placing alumina nanoparticles on the surface of our hybrid film, as shown in the inset of Fig. 7(a). The numerical simulations are performed by using finite-difference-time-domain (FDTD) method (Lumerical FDTD solutions software package) to calculate the reflectance spectra when the incident light reacts with the hybrid films. The thickness of TiN, TiAlN and Al_2O_3 layers are 50 nm, 30 nm and 30 nm, respectively. The topmost aluminium nanoparticles are periodic ellipsoids, with a period of p = 250 nm and a diameter of d in x-y plane while a height of 60 nm in z-axis. Half of the alumina nanoparticle is embedded into the topmost alumina layer.

As seen from the reflectance spectra in Fig. 7(a), unlike the metallic nanoparticles that usually exist plasmonic resonances,[3] the dielectric alumina nanoparticles do not cause additional resonance absorption, and thus the spectral profiles of the multilayer hybrid films are not obviously changed. Further, the reflectance dips at ~ 650 nm which originate from the non-trivial phase shift at TiN-TiAlN interface are insensitive to the alumina nanoparticles. However, the other reflectance dip at ~300 nm that is related to Al_2O_3-TiAlN interface slightly redshift (as indicated by the solid arrow) with the increase of particles' diameter. This may be attributed to that the bigger nanoparticles have higher effective dielectric thickness. Nevertheless, the variation of colors versus the particle sizes is fairly small, as shown by the in CIE1931 diagram in Fig. 7(b).

Supplementary Note 7: XRD and XPS measurement of laser-modified TiAlN-TiN surface

As shown in Fig. 8(a), the pristine sputtering TiAlN exhibit predominant peaks at 2*θ*=37.5° (black curve in Fig. 8a), representing a face centered cubic NaCl-type phase. Such phase is associated with high hardness, superior mechanical and tribological properties. In addition, a small amount of Al_2O_3 at 2θ=43.5° that originates from natural oxidation is also detected. The XRD spectrum of green-colored area indicates the coexistence of Al_2O_3 and TiO2 (green curve in Fig. 8a). With regard to the yellow-colored area, one can only observe TiO_2. This agrees with the cross-sectional observation in FIB-based analysis as shown in the main text (Fig. 4), which suggests that at high accumulated fluence, the oxidized TiAlN has been strongly ablated. Further, when using Ar^+ to gradually etch the laser-irradiated area and investigate the depth profile of oxygen (Fig. 8b), we find that the amount of oxygen exponentially decreases with the increase of etching depth. This suggests that the oxidation depth does not linearly increase the accumulated number of pulses, which is in agreement with the results in the main text (Fig. 3).

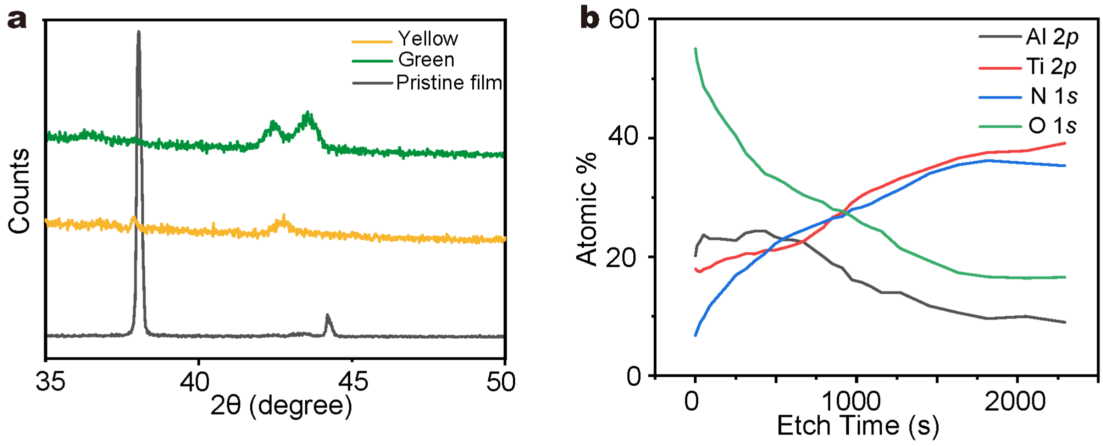

Supplementary Fig. 8 Material analysis. (a) XRD spectra of a pristine, laser-written green and yellow coloured swatch. (b) depth profile XPS analysis of element distribution of a green palette. Source data is available.

Supplementary Note 8: Repeatability tests

The printing process is highly reproducible and has high throughput. Fig. 9 shows the Academic Ring and Academic Hall of the Yungu Campus of Westlake University that was painted in 13 different colours on 64 identical Si wafers. The unpolished backsides of these 2-inch Si wafers were subsequently coated with 50 nm TiN and 60 nm TiAlN and written by our ultrafast laser. The uneven brightness originates from the uneven illumination light.

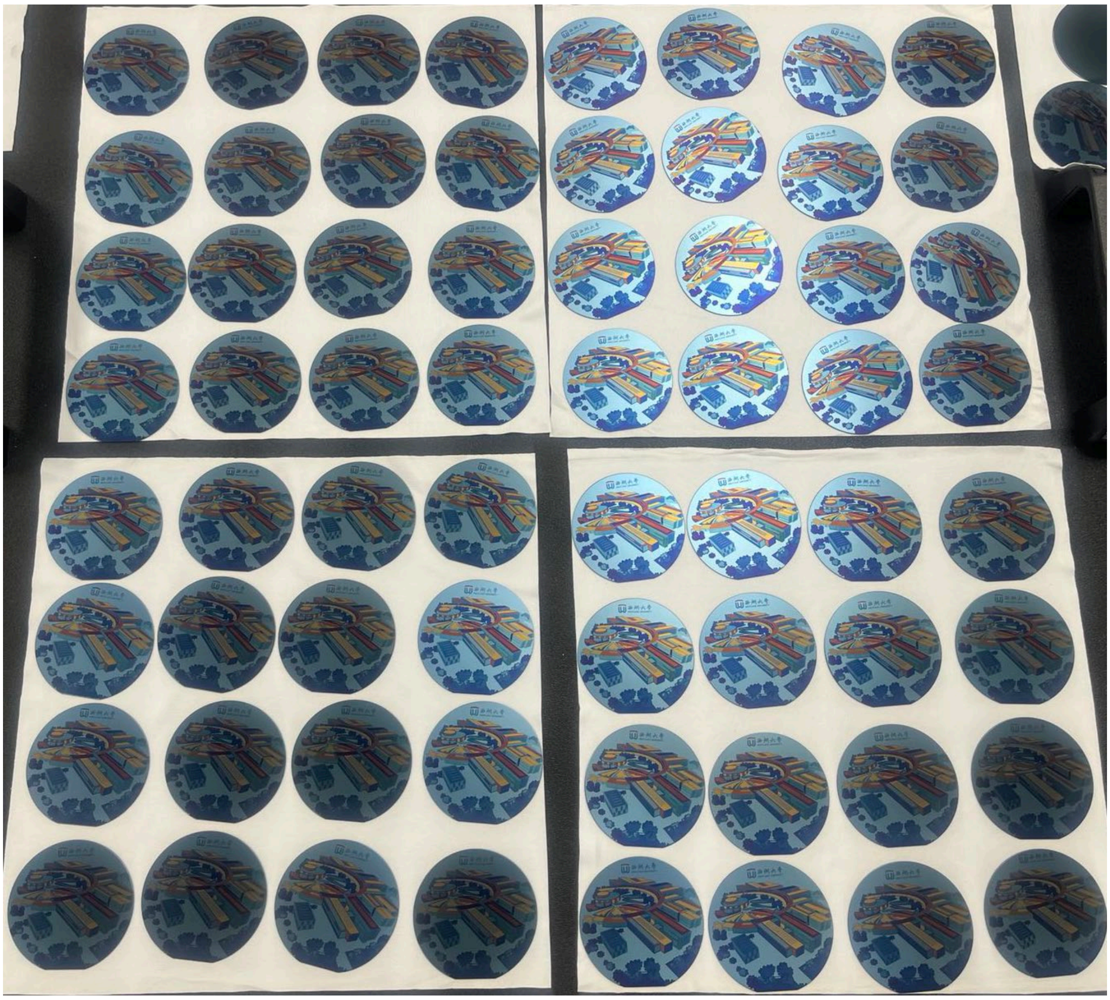

Supplementary Fig. 9 Repeatability tests. Photograph of 64 identical samples that are printed with the same laser parameters.

Supplementary Note 9: Aging tests

In addition to repeatability, durability of the laser coloring is of significance for practical applications. The color fastness tests were performed based on the measurement of color difference of an identical sample before and after aging tests. The color difference $\Delta E^*_{a,b} = \sqrt{(\Delta L)^2 + (\Delta a)^2 + (\Delta b)^2}$ was measured in Lab color space. Here ΔL represents a lightness difference between a pristine and tested sample, Δa denotes the difference in redness or greyness and Δb is blueness-yellowness differences. The aging tests include salt fog, double-85 and light bleaching experiments for 120 hours (see Methods in the main text). The caused colour differences of four different colours are plotted in Supplementary Fig. 10(a), and the corresponding photographs of referenced and tested samples are shown in Fig. 10(b-d), respectively. The color differences after all the aging tests remain $\Delta E^*_{a,b}$<7, which is an acceptable match in commercial reproduction on printing presses.[4]

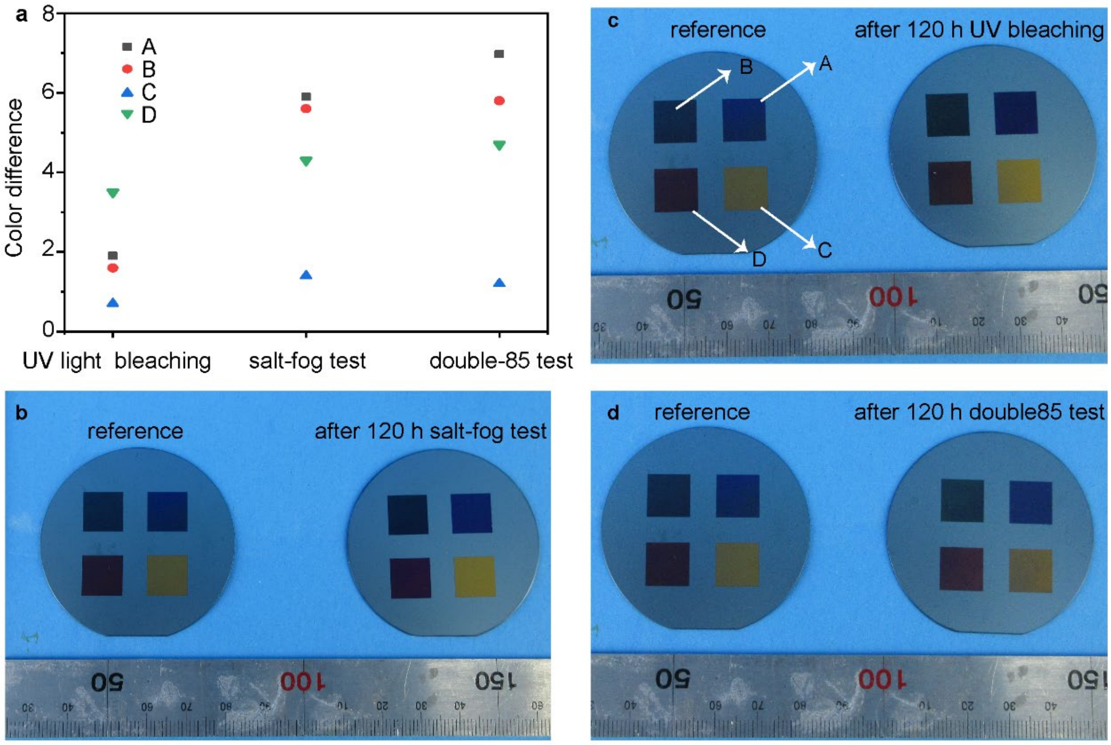

Supplementary Fig. 10 Aging tests. (a) Measured color differences of four coloured areas before and after various aging tests for 120 hours. (b-d) Photographs

of colour swatches that are used as references and after various aging tests.

Supplementary Note 10: Technology-performance indicators for laser-coloring technologies

Liu et al. recently have summarized the performances of laser coloring by pulsed lasers irradiation on solid substrates in terms of stability, viewing angle-dependences, operation cost, productivity, and resolution, including plasmonic colors from randomly self-organized metallic nanoparticles, diffractive colors from LIPSS and interfering colors from thin films including transparent oxide layer.[5] As shown in Fig. 11, the conventional laser-coloring techniques suffer either narrow gamut or viewing angle-dependent colors and moderate productivity. Our technique, relying on laser-coloring on lossy dielectric TiAlN film, exhibits a high overall performance.

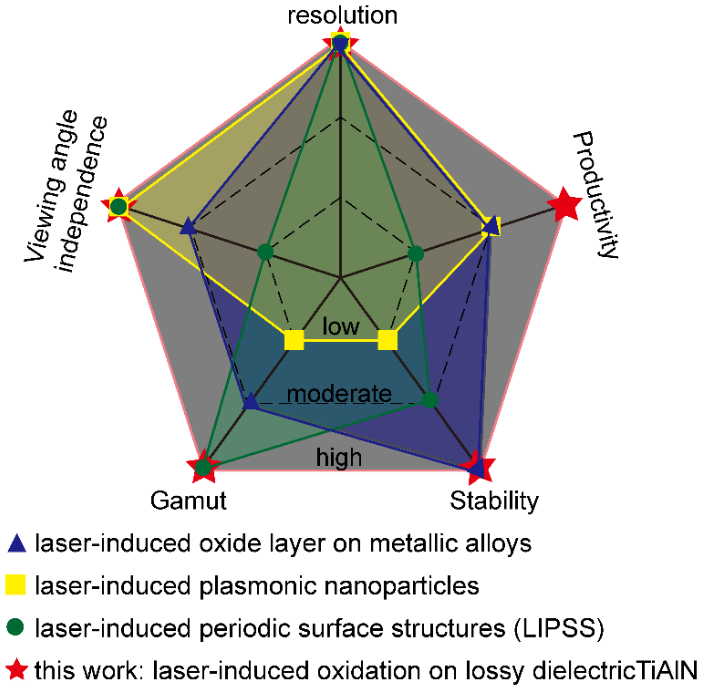

Supplementary Fig. 11 Technology performance. Technology-performance indicators expressing on low, moderate and high level that provides an overview of the state-of-the-art laser coloring techniques.

References:

1. Bonse, J.; Gräf, S. Maxwell Meets Marangoni — A Review of Theories on Laser-Induced Periodic Surface Structures, Laser & Photonics Reviews **14**, 2000215 (2020).

2. Geng, J.; Yan, W.; Shi, L.; Qiu, M. Quasicylindrical Waves for Ordered Nanostructuring, Nano Letters **22**, 9658 (2022).

3. Guay, J.-M., Cala Lesina, A., Cote, G., Charron, M., Poitras, D., Ramunno, L., Berini, P., Weck, A.: Laser-induced plasmonic colours on metals. Nature Communications **8**, 16095 (2017).

4. Antonczak, A.J., Kocon, D., Nowak, M., Koziol, P., Abramski, K.M.: Laser-induced colour marking—sensitivity scaling for a stainless steel. Applied Surface Science **264**, 229 (2013).

5. Liu, H., Lin, W., Hong, M.: Surface coloring by laser irradiation of solid substrates. APL Photonics **4**, 051101 (2019).
