# Supplementary material for: High-speed laser writing of structural colors for full-color inkless printing
Source: Nat Commun. 2023 Feb 2;14:565. doi: 10.1038/s41467-023-36275-9 (PMC9894925; doi:10.1038/s41467-023-36275-9)
Supplement: Supplementary file 3 — Description of Additional Supplementary Files [file 41467_2023_36275_MOESM3_ESM.pdf]

Description of Additional Supplementary Files:

Supplementary Movie 1: Video of laser printing process with a speed of 10 cm2/s.
